# The Greasy Pole Syndrome in *Alliaria petiolata* (Brassicaceae): The Pubescence and Wax Coverage on Stems Reduce Invasion by *Lasius niger* Ants

**DOI:** 10.3390/plants13141932

**Published:** 2024-07-13

**Authors:** Elena V. Gorb, Stanislav N. Gorb

**Affiliations:** Department of Functional Morphology and Biomechanics, Zoological Institute, Kiel University, Am Botanischen Garten 9, 24098 Kiel, Germany

**Keywords:** ant, epicuticular wax projections, *Lasius niger*, nectar thieving, running velocity, travelled distance, trichomes, visiting frequency

## Abstract

To reduce negative effects of floral visitation by ants, which do not serve as reliable cross-pollinators, some plants have developed a non-floral, stem-based defense mechanism called greasy pole syndrome. In the present study, we examined the effects of two surface features (trichomes and three-dimensional epicuticular wax coverage) on stems of *Alliaria petiolata* plants on visiting frequencies, travelled distances, and running velocities of *Lasius niger* ants. The experiments were performed with stem samples prepared from different (apical and basal) stem portions showing different surface morphologies (smooth control, covered by wax and trichomes + wax, respectively). The control, mechanically wiped stem samples lacking any surface features were significantly more often visited by ants, where they travelled significantly longer distances and moved with significantly higher velocities, compared to the intact stems. The apical and basal stem portions showed no significant differences in the measured parameters. Based on data obtained, we conclude about the main contribution of the wax to the greasy pole function of the *A. petiolata* stem via reduction of ant adhesion to the wax-bearing stem surface, whereas trichomes presumably serve as the first barrier for ants approaching usually from the ground level and protect the fragile wax coverage from an excessive deterioration.

## 1. Introduction

Compared to other Hymenoptera, which are important pollinators of angiosperms, ants are very rare pollinators despite their abundance, and the number of ant-pollinated plants is very low [1,2]. Because of their small size, which is smaller than the floral reproductive structures, usually rather smooth and hairless body surface preventing successful pollen attachment, grooming behavior leading to removal of adhered pollen before their transport, limited displacement (wingless ant workers forage mainly near their nests), and antibiotic body secretions reducing germination rates of pollen grains, ants are considered as poor agents of cross-pollination [3,4,5,6].

However, as numerous ant species are generalist foragers, they regularly visit flowers and readily consume nectar. In addition to acting as nectar thieves, i.e., removing nectar from plants without effectively pollinating their flowers, ants can cause the avoidance of flowers by reliable insect pollinators and even harass them [7,8,9,10,11,12,13,14]. Moreover, plants’ floral structures can be damaged by large mandibles of ants [15,16].

In order to reduce such negative effects of floral visitation by ants, plants have developed various defense mechanisms for deterring ants, either inside the flower (e.g., toxic nectar, ant detergents, and petal hairs [17,18,19,20]) or on non-floral plant parts (e.g., extra-floral nectaries [21,22]). The greasy pole syndrome is a non-floral defense mechanism, which is based on several morphological stem characters collectively reducing access of ants to apically situated, nectar-bearing flowers [23,24,25]. Being first described in very few plant species, such as representatives from the plant genera *Salix* (Salicaceae), *Hypenia*, and *Eriope* (both Lamiaceae), this defense mechanism was later suggested to occur much more frequently, as previously considered [26].

The features contributing to the greasy pole syndrome are slender, elongate, erect, and usually non-branched stems, where lower internodes are densely covered by rigid spreading trichomes, whereas upper internodes are glabrous, but often swelling [24,25]. Another important characteristic of such stems is the epicuticular wax coverage, composed of microscopic three-dimensional (3D) wax projections [23,24,25]. It has been assumed that the combined effect of several of these stem features hampers the access of ants to flowers and, in this way, hinders nectar-thieving: the wand-like shape causes high stem motility in windy conditions, trichomes deter ant workers from foraging at the ground level, swellings serve as additional physical barriers, whereas wax prevents ants from gaining a foothold on the stem surface.

However, the relative contribution of different stem characters in hindering access of ants is not well understood. The mostly studied feature is the epicuticular wax: its hampering effect on ants’ attachment to stem surfaces was first suggested long ago for *S. daphnoides* Vill. [23] and then observed and experimentally tested in *Eriope* plants with different ant species and in *H. vitifolia* (Pohl ex Benth.) Harley with *Lasius niger* L. (Hymenoptera: Formicidae) ants more than one century later [24,25]. This effect was explained by contamination of insect feet by dislodged wax material. In our previous experiments with tree flower stem surfaces (*Anethum graveolens* L. (Apiaceae), *Dahlia pinnata* Cav., and *Tagetes patula* L. (both Asteraceae)) and *L. niger* ants, insects covered lower distances and moved more slowly on these waxy stem samples than on control, wax-free ones [27]. The later study on the visiting frequency of *L. niger* ants on five plant species having different micro-morphologies of the upper stem surfaces, such as *Alchemilla mollis* (Buser) Rothm. (Rosaceae) with wax and trichomes, *Lilium lancifolium* Thunb. (Liliaceae) and *Salvia nemorosa* L. (Lamiaceae), both with trichomes and cuticular folds, *Tulipa gesneriana* L. (Liliaceae) with wax, and *Paeonia lactiflora* Pall. (Paeoniaceae) with smooth surface, demonstrated that *L. niger* ants avoided wax-bearing stems, in particular those without trichomes [28]. In the case of the flower stem surface of *Hippeastrum reginae* (L.) Herb. (Amaryllidaceae), it was found that its high slipperiness is based on the ease of delamination of the layered structure of single wax projections and on eventual building of the smearing surfaces [29]. Recent experiments performed with *L. niger* ants and artificial samples, mimicking flower stems of *Smyrnium rotundifolium* Mill. (Apiaceae), revealed the role of the nanostructured calcium carbonate coverage, imitating the plant epicuticular wax coverage, in the reduction of ant visits to stems [30].

In contrast, effects of other stem features contributing to the greasy pole are poorly studied and there are only limited data on these in the literature. For example, it was reported for *H. vitifolia* that *L. niger* ants introduced at the ground level became entangled in the trichomes of the basal stem part [25]. The comparison of frequencies of *L. niger* ant visits to *A. mollis, L. lancifolium*, and *S. nemorosa* flower stems showed that long and presumably soft trichomes provided appropriate attachment sites for ants in the first case, whereas stiff and rigid trichomes, which were also very variable in size and orientation, created a rather irregular and “unpredictable” surface topography that could not provide a foothold for climbing ants in the second and third plant species [28]. As for the contribution of macroscopic stem characters, there is the only experimental study demonstrating that cuff-like structures formed by upper leaves in *S. rotundifolium* served as a physical barrier against scrambling ants [30]. Ants were forced to overcome three transitions, where they could easily lose their grip, especially while moving from the vertical stem to the adaxial side of the cuff or from the adaxial to abaxial side of the cuff.

**Figure 1:**
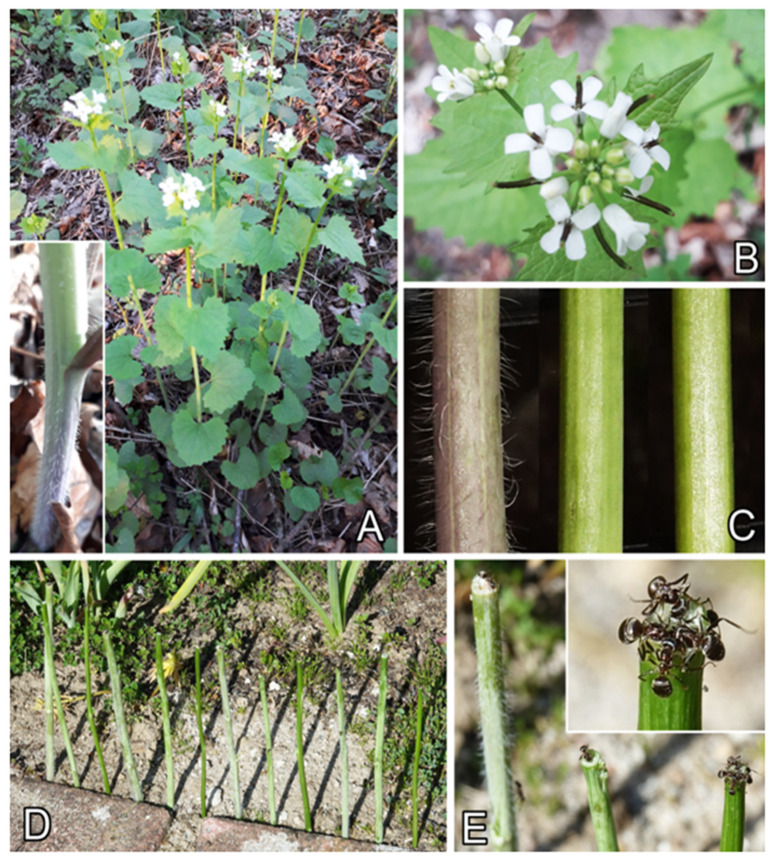
The plant *Alliaria petiolata* (**A**,**B**) and experimental set-up (**C**–**E**). (**A**) The plant in its natural environment. Inset shows the basal part of the stem, bearing trichomes. (**B**) Upper part of the plant with the apically located inflorescence. (**C**) Types of tested stem samples: the basal stem part (left), apical stem part (middle), and entirely wiped apical stem part without both 3D wax projections and trichomes (used as a control; right). (**D**) The row of 12 stem samples used in the experiment on visiting frequency. (**E**) Ants feeding on sugar syrup droplets placed on top of the tested stem samples. Inset shows the magnified top of the control stem sample.

The aim of the present study was to compare effects of two stem surface features in the garlic mustard *Alliaria petiolata* (M.Bieb.) Cavara&Grande (Brassicaceae; Figure 1A) on the stem defense function against generalist foragers of *L. niger* ants. Along with autogamy, *A. petiolata* also uses entomophily as a cross-pollination method [31], which usually ensures healthier offspring, more viable seeds, and creates new varieties. During flowering (Figure 1A,B), this plant offers freely accessible nectar to attract various insect pollinators, such as bees, flies, syrphid flies, and small beetles [31]. 

**Figure 2:**
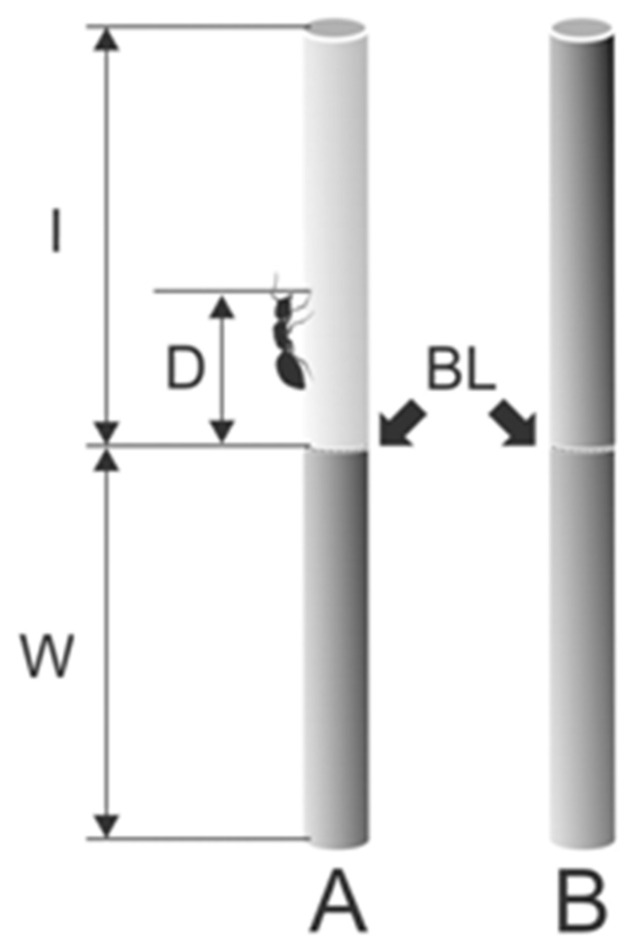
Partly (**A**) and entirely wiped (**B**) stem samples used to estimate travelled distances and running velocities of ants. BL, border line between the wiped (shown in pale gray color) and intact (shown in gray color) parts of the stem sample; I, intact stem part; D, walked distance covered by ants after they crossed the border line; W, wiped stem part. Adapted from [27]. Copyright © 2011, Springer Science Business Media B.V.

In order to prevent floral visitation by ants, which are allured by nectar and thieve it but do not serve as reliable pollinators, *A. petiolata* developed two types of stem surfaces serving to impede ants’ attachment and locomotion: (1) a pubescent surface, combined with the epicuticular wax in the basal stem part, and (2) a waxy surface in the apical stem part. To evaluate contributions of trichomes and 3D waxes to the greasy pole syndrome function, we examined visiting frequencies, measured travelled distances, and calculated running velocities of *L. niger* ants baited on the top of vertical segments of *A. petiolata* stem samples prepared from different stem portions differing in surface morphologies: (1) apical (waxes), (2) basal (waxes and trichomes), and (3) mechanically wiped apical stem portion lacking any surface features, used as a control. We performed experiments with (1) two intact and one entirely wiped stems (Figure 1C; experiment 1: visiting frequency) according to [28] and (2) two partly wiped and one entirely wiped stems (Figure 2; experiment 2: travelled distances) according to [27], and we observed the locomotory behavior of ants on different stem samples. Using cryo-scanning electron microscopy (cryo-SEM), the surface microstructure of plant surfaces was inspected. Our null hypothesis was that all the visiting frequencies, travelled distances, and running velocities of ants should be similar in all stem sample types tested in each experiment. 

## 2. Results

### 2.1. Micromorphology of Plant Surfaces

**Figure 3:**
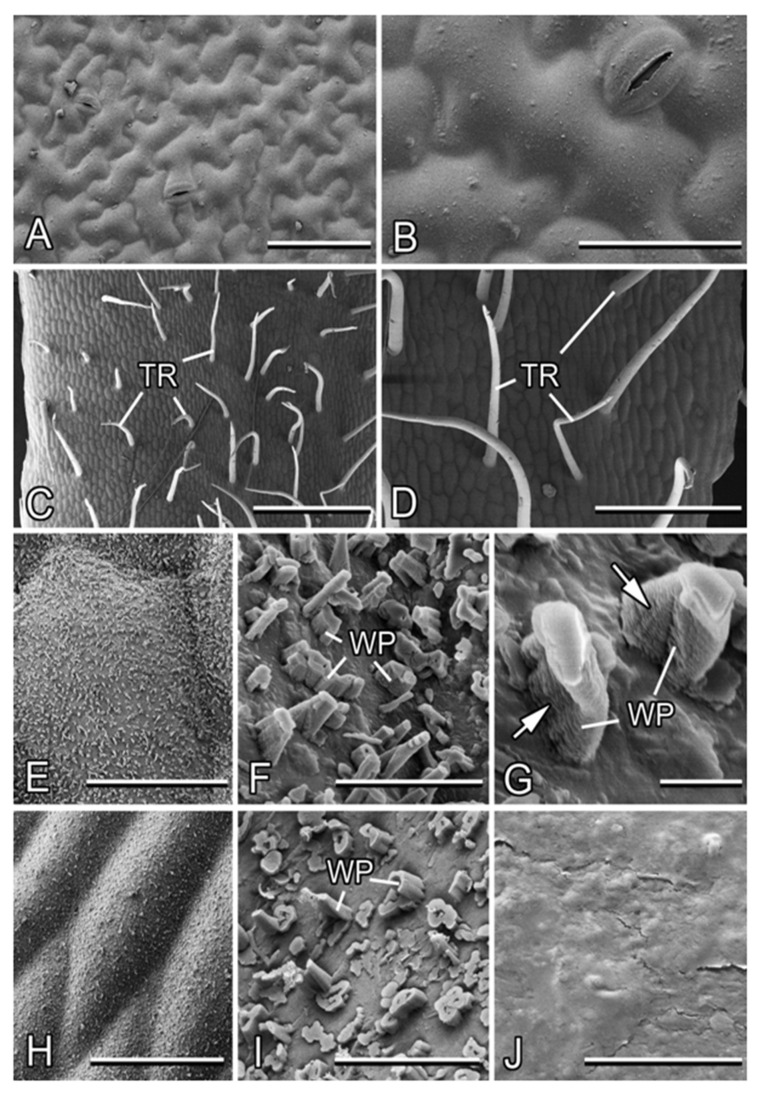
Micromorphology of the leaf and stem surfaces in *Alliaria petiolata* (cryo-SEM). (**A**,**B**) The adaxial leaf side. (**C**–**G**) The basal part of the stem. (**H**,**I**) The apical part of the stem. (**J**) The surface of the apical part of the stem after mechanical removal of the wax coverage by wiping. Arrows in (**G**) point to the ridged structure of the wax projections. Abbreviations: TR, trichome; WP, wax projections (transversely ridged rodlets). Scale bars: 1 mm (**C**), 500 µm (**D**), 100 µm (**A**), 50 µm (**B**,**H**), 40 µm (**E**), 5 µm (**F**), 4 µm (**I**,**J**), and 1 µm (**G**).

Both the adaxial and abaxial leaf sides are slightly uneven because of the somewhat convex shape of the external wall of epidermal cells (Figure 3A). The surface has a smooth appearance (Figure 3B). The only non-tabular (i.e., different from the rest) elements are bean-shaped guard cells surrounding stomata that are regularly scattered over the leaf surface (abundance: ca. 30 mm^−2^ in adaxial and ca. 200 mm^−2^ in abaxial; Figure 3A,B).

The stem shows differences in surface structure between the basal and apical parts. In the basal stem region, non-glandular, non-branched, nearly needle-shaped trichomes (length: 510 ± 129 µm, middle diameter: 33 ± 5 µm) with rounded tips (tip diameter: ca. 10 µm) pointed in different directions are regularly distributed (abundance: ca. 5–6 mm^−2^; Figure 3C,D). They lack a flexible base, readily bend or twist, and sometimes collapse. The cuticle underneath the indumentums bears a three-dimensional (3D) epicuticular wax coverage, which does not cover the surface completely and is composed of separate, transversely ridged rodlets (abundance: ca. 90 per 100 µm^2^; Figure 3E,F). These wax projections show a typical ridged structure along the longitudinal axis (Figure 3G). They have a great variety of shapes with usually irregular roundish cross-sections and are of highly variable dimensions (length: 0.9 ± 0.6 µm, cross-section: 0.5 ± 0.2 µm; Figure 3F). Some projections with larger cross-sections are hollow.

The apical stem region lacks trichomes and has only the 3D wax coverage on the cuticle surface (Figure 3H). Compared to the stem base, the transversely ridged rodlets here are more densely spaced (abundance: ca. 150 per 100µm^2^) and show even higher diversity in shapes, sizes (length: 0.4 ± 0.4 µm, cross-section: 0.7 ± 0.3 µm), and cross-sections, with the prevalence of rather short, thick, and hollow projections (Figure 3I). This makes the wax coverage more compact, but less uniform.

The squarrose trichomes create a villous surface appearance of the basal stem part (Figure 1A), whereas the wax coverage is responsible for a pale grayish bloom on the stem surface.

### 2.2. Frequencies of Ant Visits to Stem Samples

In the experiment with two intact (apical and basal) stem samples and one entirely wiped basal stem sample (experiment 1, see Section 4.3.1 and Figure 1D for the experimental set-up), stem surface characteristics strongly affected the access of ants to the top of the stems. Table S1 in the Supplementary Materials shows raw data on ant visits to each sample. In each set of counts (one set is an enumeration of ant visit to twelve test samples at a certain moment of time), at least one type of stem sample (see Figure 1C for sample types) was visited by ants. The lowest number of individual ants observed in 1 set of counts was 1 (day 7, session 1, count 1, and session 2, count 4), whereas the highest number reached 24 individuals (day 2, session 2, count 2, and day 3, session 2, count 6; Table S1). The control stem type (wiped stem samples) always had ants feeding on the syrup (with the only exception at day 3, session 1, count 1), and in 23 cases (out of 57), this was the only sample type with ants (Table S1). Altogether, as a result of 57 sets of counts, we recorded 364, 48, and 61 ant visits to the control, basal, and apical stem sample types, respectively. 

**Figure 4:**
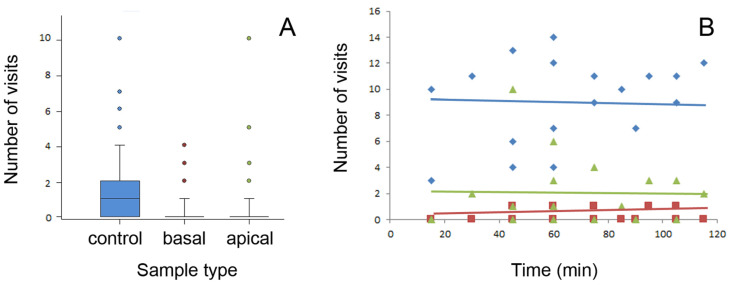
Number of ant visits per sample type registered during one count (**A**) and number of ants counted on all four samples of the same stem type during consecutive periods of the experimental time (i.e., during consecutive counts) (**B**). Boxplots in (**A**) show the interquartile range and medians, whiskers indicate the 1.5× interquartile range, and “°” points to an outlier. Lines in (**B**) are linear regressions: y = 6.830 + 0.0348x (F_1,16_ = 1.884, *p* = 0.193, ANOVA) for control, y = 0.227 + 0.0017x (F_1,16_ = 0.168, *p* = 0.687, ANOVA) for basal, and y = 1.957 + 0.0000552x (F_1,16_ = 0.000884, *p* = 0.877, ANOVA) for apical sample types. Control samples are shown in blue color(s), samples from the basal stem portion in red, and samples from the apical stem portion in green. Abbreviations: apical, sample from the apical stem portion; basal, sample from the basal stem portion; control, control sample of the wiped stem.

The average number of ant visits per stem sample type, which was obtained by division of the total number of ants that visited all samples of this sample type during 57 sets of recordings by the number of samples of this sample type, was 91 ± 19 in the wiped stems, 12 ± 5 in the basal stems, and 15 ± 9 in the apical stems (mean ± SD for n = 4). Different sample types demonstrated significant differences in the number of ant visits (χ^2^ = 182.996, d.f. = 2, *p* < 0.001). Among the sample types, the control wiped stems were more preferable to ants (control vs. basal: χ^2^ = 127.744, control vs. apical: χ^2^ = 108.913, both d.f. = 1, *p* < 0.001, Chi square test with Bonferroni correction), whereas the samples from the intact basal and apical stem parts did not differ from each other (χ^2^ = 0.927, d.f. = 1, *p* = 0.336, Chi square test with Bonferroni correction). The number of ant visits registered during one count on each sample type is shown in Figure 4A.

In Figure 4B, the number of ants recorded on each stem sample type is plotted against the experimental time. Here, only data from days 1, 3, and 4 with long experimental sessions were used. As seen from the graph, neither of the tested sample types showed any noticeable dependence of the number of ant visits on the time. Thus, similar frequencies of ant visits obtained in consecutive counts in all stem sample types indicated that there was no ant recruitment on either stem sample type within this experimental time. Probably, the food source, in the form of a minute drop of syrup, was not large enough to elicit recruitment.

### 2.3. Locomotory Behavior, Travelled Distance, and Running Velocity of Ants on Stem Samples

Experiments with two partly wiped (apical and basal) stem samples and one entirely wiped basal stem sample (experiment 2, see Section 4.3.2 and Figure 2 for experimental set-up) revealed an impact of stem surface morphology on locomotion of ants. Raw data on travelled distances of ants measured during five consecutive runs on three different stem sample types are presented in Table S2 in the Supplementary Materials. On stem samples containing intact stem regions, all or almost all (18 out of 20) ant individuals refused to cross the border between the intact and wiped sample parts during the first run in experiments on the basal and apical stem samples, respectively (Figure S1A in the Supplementary Materials). In the subsequent runs, the number of such insects decreased to six (basal stem) or five (apical stem). In the case of the control (i.e., entirely wiped) samples (see Figure 2B), only two tested ants did not passed the virtual border line during the first run and, starting from the third run, none of them stopped at the border.

**Figure 5:**
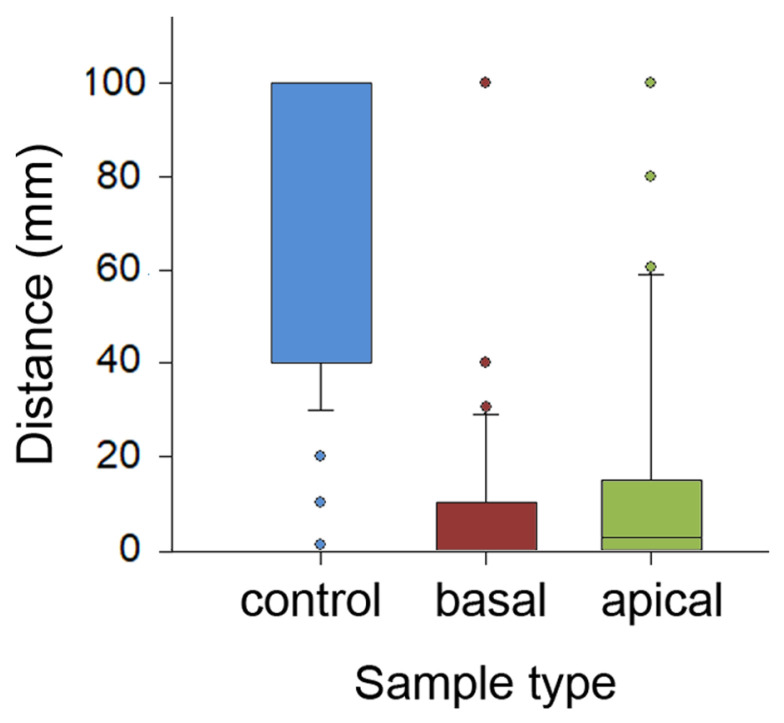
Distances covered by ants in experiments with the partly and entirely wiped stem samples. Boxplots show the interquartile range and medians, whiskers indicate the 1.5× interquartile range, and “°” points to an outlier. Abbreviations: apical, sample from the apical stem portion; basal, sample from the basal stem portion; control, control sample of the entirely wiped stem.

The comparison of travelled distances showed highly significant differences between sample types (H_2,299_ = 158.585, *p* < 0.001, Kruskal–Wallis one-way ANOVA on ranks; Figure 5). Insects covered significantly longer distances on the control, entirely wiped samples compared to samples of both other types (control vs. basal: q = 16.012; control vs. apical: q = 13.489, both *p*< 0.05, Tukey test). In the basal and apical samples, distances traversed by ants on intact sample parts were similar (q = 2.523, *p* > 0.05, Tukey test). Also, in each of five runs, travelled distances were significantly different between sample types (run 1: H_2,57_ = 44.865; run 2: H_2,57_ = 36.616; run 3: H_2,57_ = 38.682; run 4: H_2,57_ = 34179; run 5: H_2,57_ = 33.2302; all *p* < 0.001, Kruskal–Wallis one-way ANOVA on ranks; Figure S1B in the Supplementary Materials). Interestingly, in each sample type, distances gradually grew in the subsequent runs, reaching their maxima during the final 5th run (control: H_4,95_ = 33.572; basal: H_2,57_ = 20.832; apical: H_2,57_ = 25.544; all *p* < 0.001, Kruskal–Wallis one-way ANOVA on ranks). 

**Figure 6:**
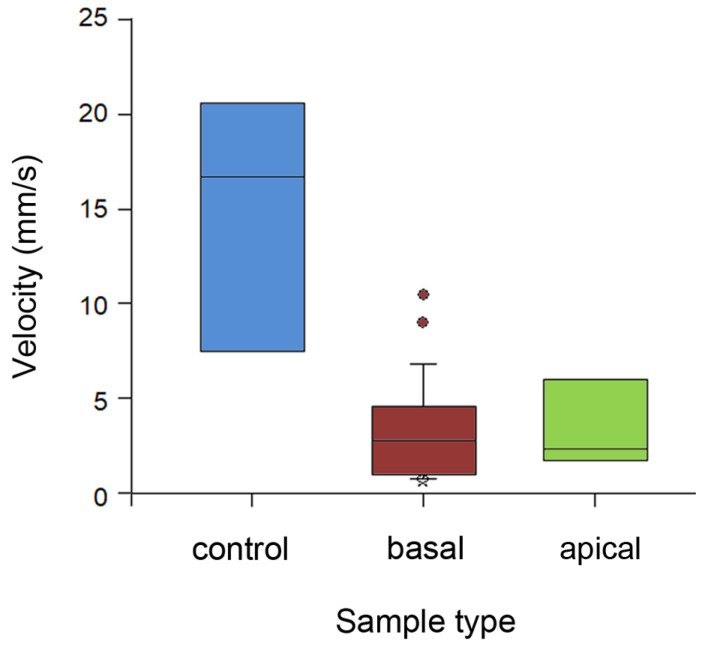
Running velocity of ants on different types of stem samples. Boxplots show the interquartile range and medians, whiskers indicate the 1.5× interquartile range, and “°” points to an outlier. Abbreviations: apical, sample from the apical stem portion; basal, sample from the basal stem portion; control, control sample of the entirely wiped stem.

Running velocity of ants differed significantly between stem types (H_2,35_ = 16.182, *p* < 0.001, Kruskal–Wallis one-way ANOVA on ranks; Figure 6). Insects ran almost 5 times faster on the wiped stems compared to both types of intact stems (control vs. basal: Q = 3.972; control vs. apical: Q = 2.546, both *p* < 0.05, Dunn’s method). On the later substrates, they demonstrated similarly lower velocity values (Q = 0.101, *p* > 0.05, Dunn’s method).

We observed that ants walked more confidently and less carefully on intact stem surfaces. Supplementary Materials Videos S1 and S2 show that ants had difficulties in trying to get a grip and, therefore, did not readily travel on these substrates. We found that ants fell down in 9%, 33%, and 35% of trials during experiments on the entirely wiped, basal, and apical stem samples, respectively. Such insects were excluded from the further experiments.

## 3. Discussion

It is well known that ants are often poor pollinators [1,2,3,4,5,6], although recently, the number of reports on ants acting as effective main or at least complementary pollinators for some plants, especially in dry or cold environments with poor quality of soil and predominance of shrub vegetation, where flying insects are not abundant, is growing [2,6,32,33]. These ant-pollinated plants are usually short and bear inflorescences close to ground level, which are composed of small and sessile flowers rich in nectar but producing either little or even no scent and a rather small quantity of pollen grains [3]. However, most entomophilic plants, which account for ca. 88% of all animal-pollinated plants, rely on bees and/or their close relatives [34], whereas using ants as a pollination agent occurs very rarely, and interactions between ants and flowers are commonly considered to be antagonistic [35]. Therefore, from the plant perspective, various floral and non-floral mechanisms, among them also stem-based greasy pole syndrome, are involved in repellence against ants, in order to decrease the rate of ants’ floral visits. 

The plant *A. petiolata* studied here displays a complex of morphological features typical for the greasy pole syndrome: slender, vertical, non-branched stems, trichomes spread over the surface of the basal stem portion, and 3D epicuticular wax covering the stem cuticle. Interestingly, transversely ridged wax rodlets composing the wax coverage on the *A. petiolata* stem were often found on shoots [27,29,36], also in combination with other types of wax projections. In the present study, we examined the effects of the surface structures (trichomes having dimensions in the sub-millimeter range and microscopic wax projections) on the stem’s deterrent function against ants. As the apical stem portion bore only waxes, whereas the basal stem portion was covered by a combination of waxes (similar to those in the apical stem part) and trichomes, this allowed us a possibility to understand the role of each type of surface feature in the stem defense function.

All the experiments performed in this study delivered complementary results and clearly demonstrated that the control wiped stem samples, lacking any surface features, were significantly more often visited by *L. lasius* ants and ensured that ants travel significantly longer distances and move with significantly higher velocities compared to the intact (i.e., covered by trichomes and/or wax projections) stems. We explain the obtained results primarily by the influence of the above plant surface structures (epicuticular wax coverage and trichomes) on the attachment and locomotory behavior of ants. The comparison between the apical waxy and the basal hairy + waxy stem portions showed no significant difference between them in either visiting frequencies, travelled distances, or running velocities of ants, although a trend toward lower values of the measured parameters in basal stems could be observed. 

It is well known that 3D epicuticular plant waxes can strongly reduce the adhesion ability and impede locomotion of non-specialized insect species, and these reports have been reviewed in [37,38,39]. Such an effect has been shown mostly as a mechanism of plant protection against insect herbivores and phytophages, whereas some insect-trapping plants, both carnivorous and ones with kettle trap flowers, employ waxes to capture and retain insect prey or pollinators, respectively (e.g., [40,41] and [42,43], respectively). The wax coverage on stems may also serve as a part of the so-called stem guard syndrome, aimed to prevent ants from visiting and supporting aphids that feed on apical stem regions [44], or greasy pole syndrome, as examined in the present study. As contributing mechanisms for reduced insect adhesion on waxy plant surfaces, the effects of the surface micro/nano-roughness (roughness hypothesis), contaminability (contamination hypothesis), absorption (fluid absorption hypothesis), and lubricating abilities (wax dissolution and separation layer hypotheses) have been previously proposed [45,46] and partially tested experimentally. Taking into account (1) the rather small dimensions and sparse distribution of wax projections, creating a pronounced micro-rough surface topography, minimizing the real contact area, and (2) the layered structure of wax projections that can presumably delaminate easily and release some exfoliated wax material, which either contaminate insects’ attachment organs and/or form a separation layer between insect pads and the plant surface, we suggest the roughness, contamination, and/or solid lubrication to be the most probable mechanisms for stem protection against ants in the case of the *A. petiolata* wax studied here. Additionally, because the transversely ridged rodlets of *A. petiolata* resemble those covering flower stems of *H. reginae*, it is plausible to assume the formation of a smeared wax layer, leading to a decreased attachment (friction) force [29]. This effect might be similar to that previously reported in *H. reginae* [29]. On the other hand, the wax coverage in *A. petiolata* is rather loose and conceivably has a low capillarity; therefore, it cannot effectively absorb insect pad secretion as distinct, for example, from the dense and thick wax coverage in *N. alata* that has experimentally revealed a strong oil adsorption ability [47]. Also, *A. petiolata* wax is probably not able to cause hydroplaning, since it is very unlikely to obtain a thick layer of fluid by dissolving such a small amount of wax material in insect adhesive fluid. 

Trichomes usually serve as physical barriers against herbivorous and phytophagous insects and can have negative impacts on insects’ body weight, behavior, performance, fitness, population dynamics, host-plant choice, and acceptance (reviewed in [48]). They are also involved in permanent and temporary entrapment of insects by plants [40,42,43,49] and in stem protection syndromes against ants [24,25,28,44]. The effects of trichomes depend on their shape, size, abundance, mechanical properties, and presence of secretions [28,48,49]. In the basal portion of *A. petiolata* stem, sparsely distributed, sub-millimeter-long, non-branched trichomes, pointed in different directions, formed a rather unpredictable surface topography. Although bending and twisting readily, these trichomes are not very soft and flexible and could not guarantee appropriate anchorage sites for ant claws (in contrast to previously tested long and soft trichomes on *A. mollis* flower stem [28]). Therefore, we did not observe better performance of ants on this stem sample type in either of the performed experiments. These results are in line with our previous data obtained with *L. lancifolium* and *S. nemorosa* flower stems covered by rather stiff and rigid trichomes [28]. Thus, contrary to some other described examples, where trichomes improved the surface quality of waxy plant substrates for insect attachment (e.g., [45,48,50,51]), the presence of trichomes did not affect the impact of waxes, but only non-significantly influenced the measured experimental parameters compared to those obtained on the apical, solely wax-bearing part of *A. petiolata* stem. Taking into consideration the fragile nature of the wax coverage studied, we suppose that the rather robust pubescence on the lower stem portion, while serving as the first barrier for ants approaching usually from the ground, also protects the underlying wax from an excessive deterioration.

The findings that (1) the intact stem surface samples were still visited by some ants (in the visiting frequency experiment), and (2) the number of ants refused to cross the border between the wiped and intact stem parts decreased and the travelled distances on the intact stem surface samples increased in the sequential trials, support our previous hypothesis that ants are, in general, capable of walking on such demanding substrates [27,28]. Since in our experiments with intact plant surfaces ants had no other choice (see the experimental set-up description in Section 4.3.2), they just needed a certain time to adapt their walking method, because climbing on these challenging stems presumably required additional locomotory efforts. In nature, where generalist ants usually have a large choice of diverse plants, they prefer plant stems with “good” surface quality for attachment, i.e., those lacking obstacles or slippery coverages.

## 4. Materials and Methods

### 4.1. Plant and Insect Species

The garlic mustard *A. petiolata* is an herbaceous biennial plant. It forms a rosette of round green leaves close to the ground in the first year and develops into a mature flowering plant the following spring. In the second year, plants reach 30–120 cm in height and have cylindrical stems, showing a week pubescence at the basal region and bearing alternately arranged, stalked, triangular leaves and apically located, small racemose inflorescences composed of white flowers (Figure 1A,B) [31,52]. The flowers possess freely accessible nectar and, after self-fertilization or cross-pollination by various small insects, such as bees, flies, syrphid flies, or even beetles, produce siliques [31].

*Alliaria petiolata* is a forest plant growing in humid habitats. It is native for most parts of Europe, and Central Asia to North Myanmar, and is also found in some places in North Africa [53]. *A. petiolata* is naturalized in North America and South America and is considered as an invasive plant [54].

Plants for both cryo-SEM examination and experiments were collected in the wood along the walking path in the vicinity of the biotop Domaenetal near Kronshagen (district Rendsburg-Eckernfoerde, federal state Schleswig-Holstein, Germany; 54.331636° N, 10.085066° E).

The black garden ant *L. niger* is an omnivorous species with workers also foraging routinely on various plants [55]. They can thieve nectar from flowering plants and collect honeydew from aphids living on plants. Being native to South America and Africa [56], this ant species is newly distributed in Europe at places of human disturbance, such as roadsides, gardens, etc.

**Figure 7:**
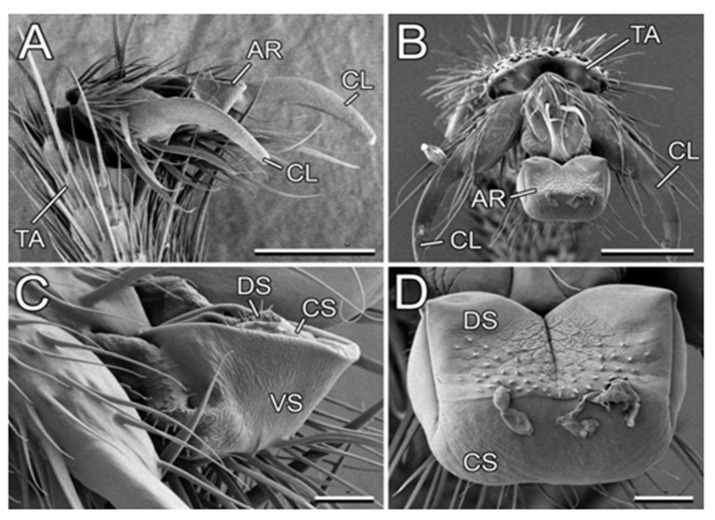
Attachment system in the *Lasius niger* ant (cryo-SEM). (**A**,**B**) Terminal tarsomere and pretarsus bearing claws and the arolium in a folded (**A**) and spread condition (**B**). (**C**,**D**) Arolium in a folded (**C**) and spread condition (**B**). AR, arolium; CL, claws, CS, contact surface of the arolium; DS, dorsal side of the arolium; TA, tarsomere; VS, ventral side of the arolium. Scale bars: 50 µm (**A**,**B**) and 10 µm (**C**,**D**). Adapted from [27]. Copyright © 2011, Springer Science Business Media B.V.

In our study, we used *L. niger* as a model generalist ant species because (1) according to our personal observation, it is associated with diverse plant species, and (2) it was available in a great number at the study site. Its tarsal attachment organs, represented on each foot by paired claws and a smooth adhesive pad (arolium; Figure 7), are described in detail by Gorb and Gorb [27].

### 4.2. Microscopy

Plant material (upper leaves and basal and apical portions of a stem) was taken from living plants. Small samples (1 cm × 1 cm for leaves and 1 cm-long for stems) were cut out from middle regions of the leaves and stem portions and gripped in a small vice on a metal holder. The preparations were transferred into a cryo-stage preparation chamber (Gatan ALTO 2500 Cryo Preparation System, Gatan Inc., Abingdon, UK), frozen at −140 °C, and sputter-coated with gold–palladium (thickness 6 nm). Surfaces of the upper (adaxial) and lower (abaxial) leaf sides and of both basal and apical stem portions were examined in a frozen condition (−120 °C) in a cryo-SEM Hitachi S-4800 (Hitachi High-Technologies Corporation, Tokyo, Japan) at 3 kV. Trichomes and waxes were described, and their types were identified according to Voigt et al. [48] and Barthlott et al. [36], respectively. From the digital images obtained with cryo-SEM, morphometrical variables of trichomes and wax projections were measured using the image analysis software SigmaScan Pro 5 (SPSS Inc., Chicago, IL, USA). These data are presented in the text as mean ± SD for n = 10.

### 4.3. Experiments

The experiments were carried out in a private garden (Kronshagen) with freshly prepared, ca. 15 cm-long stem samples of *A. petiolata*, where leaves were cut out. We used three types of stem samples: (1) intact basal portion, (2) intact apical portion, and (3) mechanically wiped apical portion, used as a control (Figure 1C). Tests were performed in May, when *A. petiolata* plants were flowering, at a temperature of 18–20 °C and 40–65% relative humidity.

#### 4.3.1. Experiment I: Visiting Frequency

Four stem samples of each type (twelve samples in total) were dug into the soil at a ca. 2 cm depth. The samples were placed abreast in a random order at a 3 cm distance from the neighboring sample (Figure 1D), at a ca. 20 cm distance from the nearest ant nest. We placed a 15 µL droplet of a custom-prepared sugar syrup (weight of double-distilled water to sugar was 1:1) on the tip of each stem sample and, after 30 min, individually checked each single sample and recorded the number of ants feeding on the droplet (Figure 1E). Afterwards, we counted ants every 10–15 min. The syrup droplets were renewed after each counting of ants to maintain a similar amount of syrup independently of the number of visiting ants. 

We conducted the experiment during eight days in the morning (9:00–12:30), afternoon (15:00–18:00), and/or evening (18:00–20:30) in similar weather conditions. In each experimental session (means for one day, either in the morning, afternoon, or evening), we used new stem samples and changed the position of different sample types. On average, there were four sets of recordings (1 set was an enumeration of ant visits to 12 test samples at a certain moment of time) per session. Altogether, 57 sets of counts were performed, and 473 ant visits were recorded.

Data on the number of ant visits in the three stem sample types were analyzed using the Chi square test (SigmaStat 3.5, SPSS Inc., Chicago, IL, USA). For pairwise comparisons between different sample types, we used the Chi square test with Bonferroni correction ($\alpha =\frac{0.05}{3}=0.017$). In all tests, the expected frequency in each sample type was calculated as expected = total number of recorded ants/number of sample types.

#### 4.3.2. Experiment II: Locomotory Behavior of Ants

In 15 cm-long samples representing the intact basal and apical stem portions (types 1 and 2 in Section 4.3, respectively), the lower (5 cm-long) region of the sample was wiped free of either both trichomes and waxes (basal stem sample) or just waxes (apical stem sample; Figure 2A). Thus prepared samples together with the completely wiped (type 3, control) sample (Figure 2B) were placed vertically, with their upper ends fixed to the supports and lower ends freely hanging in the air.

We executed the experiments with ants, as described in [27], where we observed the locomotory behavior and recorded the maximum travelled distances of individual ants on stem samples with different surface structures. However, distinct from the above study, here, intact regions were 10 cm in length and not only wax-bearing (apical stem sample) but also hairy combined with wax in the basal stem sample. In these sample types, borders between the wiped and intact regions were clearly visible. For the entirely wiped samples, we considered a virtual border between the lower (5 cm-long) and upper (10 cm-long) stem parts.

We examined 4 samples of each stem sample type (altogether 12 samples) and tested 20 individual ants (with 5 consecutive runs each) on each sample type. In total, 60 ant individuals were screened, and 300 runs were analyzed.

Data on travelled distances were compared between different sample types and different ant runs using non-parametric Kruskal–Wallis one-way ANOVA on ranks (SigmaStat 3.5, SPSS Inc., Chicago, IL, USA), followed by the post hoc Tukey test for the pairwise comparisons.

Additionally, the behavior of ants on test samples during the experiment was video-recorded (25 frames per second) with the videocamera Sony RX10 (Sony Corporation, Tokyo, Japan). Here, 27 obtained videosequences were edited using the free video-editing software Avidemux 2.7.4 (available at “http://avidemux.sourceforge.net (accessed on 21 May 2020)”) and analyzed with the free test version of the on-screen measuring tool PixelStick 2.16.2 (Plum Amazing Essential Software, available at “https://plumamazing.com/product/pixelstick/ (accessed on 21 May 2020)”). The running velocity of insects, *V*, was calculated from 760 ms to 37.2 s long video sections (8 for the wiped stems, 25 for the intact basal stems, and 4 for the intact apical stems), embracing successful locomotion as follows:

$$V=\frac{D}{Te-Ts},$$

where *D* is the travelled distance (mm) measured from video sections, *Te* is the end time (*s*), and *Ts* is the start time (*s*). 

Data on running velocity were analyzed with non-parametric Kruskal–Wallis one-way ANOVA on ranks (SigmaStat 3.5, SPSS Inc., Chicago, IL, USA), followed by the post hoc Dunn’s method for pairwise comparisons between samples.

## Data Availability

Data are contained within the article and Supplementary Materials. Further raw data will be made available by the authors upon request.

## Supplementary Materials

The following supporting information can be downloaded at: https://www.mdpi.com/article/10.3390/plants13141932/s1. Table S1: Number of *Lasius niger* ant individuals registered on each sample (1–4) of the three sample types (control wiped, intact basal, and intact apical) prepared from *Alliaria petiolata* stems at the time of 57 visits of experimenters during 8 days (20 experimental sessions). Table S2: Distances (in cm) traversed by *Lasius niger* ants during 5consecutive runs on intact parts of the partly wiped samples of the basal and apical sample types and on the control entirely wiped sample type prepared from *Alliaria petiolata* stems (20 ant individuals for each sample type, with 4samples each). Figure S1: Number of ants that refused to cross the border between the wiped and intact stem parts (**A**) and distances covered by ants in experiments with the partly and entirely wiped stem samples (**B**). Bars correspond to different sample types: control stem type (blue), basal stem type (red), and apical stem type (green). Video S1: Behavior of *Lasius niger* ants on the partly wiped sample prepared from the basal stem portion of *Alliaria petiolata* plant. Video S2: Behavior of *Lasius niger* ants on the partly wiped sample prepared from the apical stem portion of *Alliaria petiolata* plant.
