# Supplementary material for: The Greasy Pole Syndrome in Alliaria petiolata (Brassicaceae): The Pubescence and Wax Coverage on Stems Reduce Invasion by Lasius niger Ants
Source: Plants (Basel). 2024 Jul 13;13(14):1932. doi: 10.3390/plants13141932 (PMC11280409; doi:10.3390/plants13141932)
Supplement: Supplementary file 1 [file plants-13-01932-s001.zip › Table S2.pdf]

DISTANCES

ant no.	control					basal					apical				
	run 1	***run2***	***run3***	***run4***	***run5***	***run1***	***run2***	***run3***	***run4***	***run5***	***run1***	***run2***	***run3***	***run4***	***run5***
ant 1	40	40	70	80	60	0	0	5	10	30	0	10	10	0	40
ant 2	80	80	100	100	100	0	0	5	7	0	0	15	40	100	100
ant 3	0	40	40	100	100	0	0	4	0	10	0	10	20	100	100
ant 4	40	100	100	100	80	0	30	40	0	0	0	0	0	10	10
ant 5	30	80	100	100	100	0	0	5	5	0	0	20	10	0	10
ant 6	100	100	50	30	100	0	0	0	0	0	0	5	0	0	15
ant 7	30	30	80	30	100	0	0	10	0	20	10	5	0	5	0
ant 8	30	100	30	30	100	0	0	0	5	30	0	0	0	5	10
ant 9	30	40	30	80	100	0	0	5	10	20	5	0	10	100	100
ant 10	0	0	100	100	100	0	10	20	0	0	0	10	10	10	20
ant 11	30	30	100	100	100	0	20	20	20	0	0	20	30	60	50
ant 12	30	30	100	100	100	0	0	10	30	0	0	0	0	5	0
ant 13	30	80	40	100	100	0	0	10	0	0	0	0	0	5	0
ant 14	100	100	80	100	100	0	10	0	0	0	0	0	0	0	10
ant 15	100	100	100	100	100	0	0	30	30	100	0	0	0	10	0
ant 16	50	100	100	100	100	0	0	0	0	40	0	0	0	0	0
ant 17	40	50	100	100	100	0	10	10	10	30	0	10	0	30	80
ant 18	20	50	60	100	100	0	0	0	0	0	0	5	30	20	100
ant 19	10	50	50	100	100	0	0	5	1	2	0	0	30	0	100
ant 20	10	100	70	100	100	0	0	0	5	0	0	0	0	30	40
