# Supplementary material for: The Greasy Pole Syndrome in Alliaria petiolata (Brassicaceae): The Pubescence and Wax Coverage on Stems Reduce Invasion by Lasius niger Ants
Source: Plants (Basel). 2024 Jul 13;13(14):1932. doi: 10.3390/plants13141932 (PMC11280409; doi:10.3390/plants13141932)
Supplement: Supplementary file 1 [file plants-13-01932-s001.zip › Table S1.pdf]

VISITS

day	session	daytime	count	control				basal				apical			
				1	***2***	***3***	***4***	***1***	***2***	***3***	***4***	***1***	***2***	***3***	***4***
1	1	evening	1	2	0	1	1	0	0	0	0	0	0	0	0
			2	2	0	0	2	0	0	0	0	0	0	0	0
			3	4	1	0	6	0	0	0	0	0	0	0	0
			4	1	0	0	6	0	0	0	0	0	0	0	0
			5	1	0	0	10	0	0	0	0	0	0	0	0
			6	1	2	0	3	0	0	0	0	0	0	0	0
			7	3	6	3	6	0	0	0	0	0	0	0	0
2	1	morning	1	0	4	3	3	0	0	0	0	0	0	0	0
	2	evening	1	3	3	2	3	1	0	0	0	5	0	0	0
			2	5	5	5	0	4	3	0	0	2	0	0	0
3	1	morning	1	0	1	1	1	0	0	0	0	0	0	0	0
			2	7	2	0	3	0	0	1	0	0	0	1	0
			3	0	2	2	1	1	0	3	1	1	0	2	0
	2	afternoon	1	1	0	3	0	0	1	1	0	0	0	0	1
			2	1	3	4	5	2	1	0	0	0	0	0	0
			3	2	1	4	2	2	2	2	4	0	0	3	0
			4	1	0	5	1	2	1	1	1	0	0	1	0
			5	2	2	1	0	0	0	0	0	0	0	0	0
			6	2	0	5	6	1	0	0	0	0	0	0	10
			7	4	1	7	2	0	0	1	0	1	0	0	5
3	1	morning	1	0	0	0	0	0	0	0	0	1	1	0	1
4	1	afternoon	1	2	3	3	2	0	0	0	0	0	0	0	0
			2	2	2	1	6	0	0	0	0	1	0	0	1
			3	0	2	2	2	1	0	0	0	0	0	1	0
			4	3	2	0	2	0	0	0	0	1	0	1	1
			5	3	1	2	3	0	0	1	0	1	0	2	1
			6	3	2	2	3	0	0	0	0	0	0	0	1
			7	3	2	3	3	0	0	1	0	1	0	0	2
	2	evening	1	3	1	3	2	0	0	1	0	1	0	0	2
			2	5	1	1	5	0	0	0	0	1	0	0	1
			3	3	1	1	0	1	0	0	0	3	2	1	0
5	1	morning	1	0	1	1	0	1	1	1	1	0	0	0	0
6	1	afternoon	1	1	0	1	4	0	0	0	0	0	0	0	0
			2	2	0	0	2	0	0	0	0	0	0	0	0
			3	1	0	2	1	0	0	0	0	0	0	0	0
7	1	morning	1	1	0	0	0	0	0	0	0	0	0	0	0
	2	afternoon	1	1	1	0	1	1	0	0	0	0	0	0	0
			2	1	0	4	0	0	0	0	0	0	0	0	0
			3	1	1	1	1	0	0	0	0	0	0	0	0
			4	0	1	0	0	0	0	0	0	0	0	0	0
			5	0	0	4	0	0	0	0	0	0	0	0	0
			6	0	0	2	1	0	0	0	0	0	0	0	0
	3	evening	1	1	0	1	0	0	0	0	0	0	0	0	0
			2	1	0	1	0	0	0	0	0	0	0	0	0
			3	1	0	1	0	0	0	0	0	0	0	0	0
			4	1	2	0	0	0	0	0	0	0	0	0	0
8	1	evening	1	1	0	0	2	1	0	0	0	0	0	0	0
			2	1	0	1	3	0	0	0	0	0	0	0	0
			3	3	0	2	3	0	0	0	0	0	0	0	0
			4	1	0	1	1	0	0	0	0	0	0	0	0
			5	0	3	0	0	0	0	0	0	0	0	0	0
			6	2	2	0	0	0	0	0	0	0	0	0	0
			7	2	2	0	1	0	0	0	0	0	0	0	0
			8	2	1	1	1	0	0	0	0	0	0	0	0
			9	3	0	1	0	0	0	0	0	0	0	0	0

		10	1	1	0	1	0	0	0	0	0	0	0	0
		11	0	2	0	1	1	0	0	0	0	1	0	0
