# Supplementary figures and images for: The Greasy Pole Syndrome in Alliaria petiolata (Brassicaceae): The Pubescence and Wax Coverage on Stems Reduce Invasion by Lasius niger Ants
Source: Plants (Basel). 2024 Jul 13;13(14):1932. doi: 10.3390/plants13141932 (PMC11280409; doi:10.3390/plants13141932)

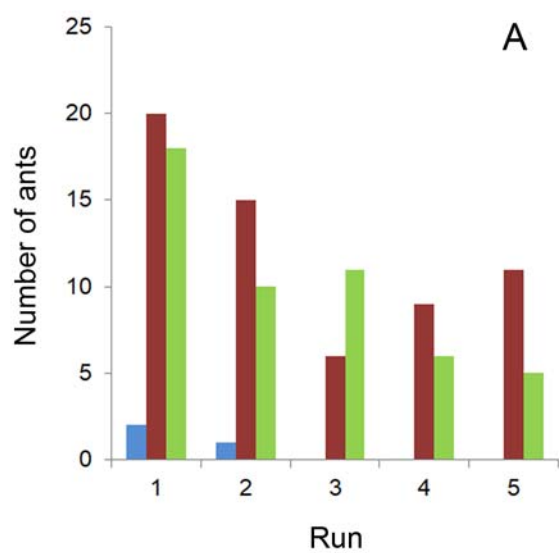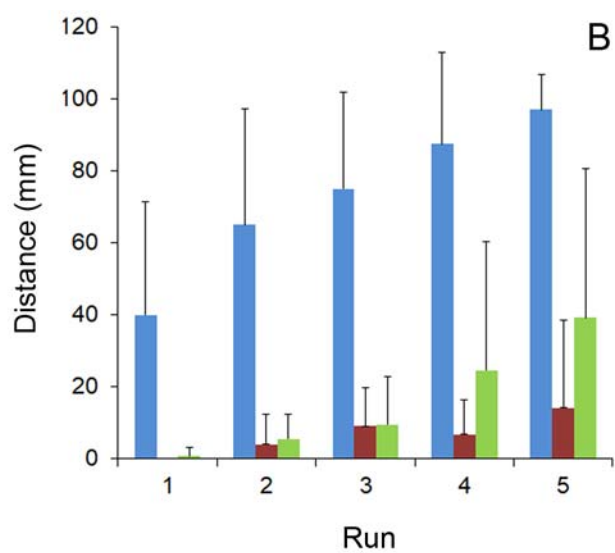

Supplement: Supplementary file 1 [file plants-13-01932-s001.zip › Figure S1.pdf]
